# Investigation of *Campylobacter concisus* gastric epithelial pathogenicity using AGS cells

**DOI:** 10.3389/fmicb.2023.1289549

**Published:** 2024-01-11

**Authors:** Christopher Yau Man Luk, Seul A. Lee, Nicholas Naidovski, Fang Liu, Alfred Chin Yen Tay, Liang Wang, Stephen Riordan, Li Zhang

**Affiliations:** ^1^School of Biotechnology and Biomolecular Sciences, University of New South Wales, Sydney, NSW, Australia; ^2^Helicobacter Research Laboratory, School of Pathology and Laboratory Medicine, Marshall Centre for Infectious Diseases Research and Training, University of Western Australia, Perth, WA, Australia; ^3^Laboratory Medicine, Guangdong Provincial People’s Hospital (Guangdong Academy of Medical Sciences), Southern Medical University, Guangzhou, Guangdong, China; ^4^The Center for Precision Health, School of Medical and Health Sciences, Edith Cowan University, Perth, WA, Australia; ^5^Department of Medical Informatics, School of Medical Informatics and Engineering, Xuzhou Medical University, Xuzhou, Jiangsu, China; ^6^Gastrointestinal and Liver Unit, Prince of Wales Hospital, University of New South Wales, Sydney, NSW, Australia

**Keywords:** *Campylobacter concisus*, *Campylobacter*, gastritis, oral *Campylobacter*, CYP1A1

## Abstract

*Campylobacter concisus* is an oral bacterium. Recent studies suggest that *C. concisus* may be involved in human gastric diseases. The mechanisms, however, by which *C. concisus* causes human gastric diseases have not been investigated. Here we examined the gastric epithelial pathogenicity of *C. concisus* using a cell culture model. Six *C. concisus* strains and the human gastric epithelial cell line AGS cells were used. IL-8 produced by AGS cells after incubation with *C. concisus* was measured using enzyme-linked immunosorbent assay (ELISA), and AGS cell apoptosis was determined by caspase 3/7 activities. The effects of *C. concisus* on actin arrangement in AGS cells was determined using fluorescence staining. The effects of *C. concisus* on global gene expression in AGS cells was determined by transcriptomic analysis and quantitative real-time PCR (qRT-PCR). The role of the upregulated *CYP1A1* gene in gastric cancer survival was assessed using the Kaplan-Meier method. *C. concisus* induced production of IL-8 by AGS cells with strain variation. Significantly increased caspase 3/7 activities were observed in AGS cells incubated with *C. concisus* strains when compared to AGS cells without bacteria. *C. concisus* induced actin re-arrangement in AGS cells. *C. concisus* upregulated 30 genes in AGS cells and the upregulation of *CYP1A1* gene was confirmed by qRT-PCR. The Kaplan-Meier analysis showed that upregulation of *CYP1A1* gene is associated with worse survival in gastric cancer patients. Our findings suggest that *C. concisus* may play a role in gastric inflammation and the progression of gastric cancer. Further investigation in clinical studies is warranted.

## Introduction

*Campylobacter concisus* is a gram-negative bacterium that is motile, with a curved or spiral shape. The bacterium can grow under both anaerobic and microaerophilic conditions, with hydrogen gas being crucial for its growth (Lee et al., 2014). *C. concisus* is further classified into two genomospecies (GS): GS1 and GS2, distinguished by the core-genome, 23 rRNA gene, and GS-specific genes (Miller et al., 2012; Chung et al., 2016; Huq et al., 2017; Liu et al., 2018; Aagaard et al., 2021; Cornelius et al., 2021). Previous studies have reported that *C. concisus* GS2 strains exhibit better adaptation in the gastrointestinal tract (Wang et al., 2017) and a better ability to invade intestinal epithelial cells compared to GS1 strains (Kalischuk and Inglis, 2011; Ismail et al., 2012; Mahendran et al., 2015). These findings align with the observation that GS2 strains are more frequently detected in mucosal biopsy samples and fecal samples from patients with gastrointestinal diseases (Kirk et al., 2018). Virulence factors of *C. concisus*, such as zonula occludens toxin, phospholipase A, as well as the functional protein BisA, have been characterized (Istivan et al., 2004; Mahendran et al., 2016; Benoit and Maier, 2023).

While commonly present in the human oral cavity as a commensal bacterium (Zhang et al., 2010), *C. concisus* is associated with inflammatory conditions of extraoral diseases such as inflammatory bowel disease (IBD), including Crohn’s disease (CD) and ulcerative colitis (UC) (Zhang L. et al., 2009; Man et al., 2010; Mukhopadhya et al., 2011), microscopic colitis, and Barrett’s esophagus (Macfarlane et al., 2007; Yang et al., 2009; Nielsen et al., 2020). Previous studies have also investigated the pathogenic mechanisms by which *C. concisus* may contribute to the development of the associated intestinal and esophageal diseases, including the induction of proinflammatory cytokines such as interleukin 8 (IL-8) and tumor necrosis factor alpha (TNF-α), epithelial cell death, immunmodulators MD-2 and programmed death-ligand 1 (PD-L1), as well as enhancing the responses of epithelial cells and macrophages to commensal bacterial species (Lee et al., 2021).

Recent studies suggest that *C. concisus* may also be involved in human gastric diseases. A study by Ferreira et al. (2022) examined the cultivation of *Helicobacter pylori* and *C. concisus* from 2,191 gastric biopsies. They reported that *C. concisus* was cultured from 50 gastric biopsies (50/2191, 2.3%) and *H. pylori* cultured from 168 gastric biopsies (168/2191, 7.7%). In twenty-eight cases with concurrent histology, *C. concisus* was found to be *H. pylori* immunoreactive positive (Ferreira et al., 2022). A study by Cui et al. (2019) examined the tongue coating microbiome of 78 patients with gastritis and 50 healthy controls. This study found that the abundance of *C. concisus* in tongue coating microbiome was associated with the gastric precancerous cascade. They also detected *C. concisus* in gastric fluids of patients with gastritis (Cui et al., 2019).

Despite being suggested to play a role in human gastric diseases, no studies have examined the mechanisms by which *C. concisus* may contribute to the pathogenesis of gastric diseases. Considering that gastric epithelial cells are the first line of human cells to encounter pathogens in the stomach, this study aimed to examine the pathogenic effects of *C. concisus* strains on human gastric epithelial cells using a cell culture model. Our data provide novel insights into understanding *C. concisus* gastric pathogenicity.

## Materials and methods

### Bacterial strains used in this study

For this study, we randomly selected six oral *C. concisus* strains with complete genomes sequenced from *C. concisus* strains we previously isolated from human saliva samples (Liu et al., 2020). P10CDO-S2, P3UCO1, and H1O1 are GS1 strains and P2CDO4, P15UCO-S2, and H16O-S1 are GS2 strains. The details of the six *C. concisus* strains were provided in Supplementary Table 1. The *C. concisus* strains were cultured on horse blood agar (HBA) plates, using blood agar base No. 2 (Thermo Fisher Scientific, CA, USA), supplemented with 6% defibrinated horse blood. The cultures were incubated at 37*^o^*C under anaerobic conditions with 5% hydrogen for 48 h, as previously described (Lee et al., 2014).

*H. pylori* strain 26695, a human gastric pathogen, was used as a positive control in this study (Ashktorab et al., 2008; Fazeli et al., 2016). *H. pylori* strain 26695 was cultured on HBA plates at 37*^o^*C under microaerobic conditions generated using CampyGen 2.5L Atmosphere Generation System (Thermo Fisher Scientific) for 48 h before being used in experiments.

### Maintenance of AGS cells

The human gastric adenocarcinoma cell line AGS (ATCC No. CRL-1739) was used as a model for human gastric epithelium. AGS cells were maintained in F-12K medium (Thermo Fisher Scientific) supplemented with 10% fetal bovine serum (FBS) (Cytiva, MA, USA), 100 U/mL penicillin, and 100 μg/mL streptomycin (Thermo Fisher Scientific), which was referred to as F-12K/FBS/Antibiotics medium in this study. The AGS cells were incubated in a humidified incubator at 37°C with 5% CO_2_, following recommended maintenance procedures by ATCC.

### Measurement of IL-8 by enzyme-linked immunosorbent assay (ELISA)

Enzyme-linked immunosorbent assay was used to measure IL-8 production by AGS cells in response to *C. concisus*. AGS cells were seeded on a 96-well plate at a concentration of 1 × 10^5^ cells/well in F-12K/FBS/Antibiotics medium. Following 24 h incubation, the cell culture medium was replaced with F-12K medium supplemented with FBS but without penicillin and streptomycin, which was referred to as F-12K/FBS medium. The AGS cells were then incubated with the six *C. concisus* strains (in triplicates) described above at a multiplicity of infection (MOI) of 100 for 24 h. As *C. concisus* at MOI 100 induced the production of IL-8 by other epithelial cells of the gastrointestinal tract in a previous study, this condition was therefore used in the current study (Lee et al., 2021). AGS cells without bacterial infection served as the negative control. *H. pylori* strain 26695 was also introduced at MOI 10 and 100, which served as a positive control (O’Hara et al., 2006; Zhang Y. et al., 2009). To investigate the combined effects of *C. concisus* and *H. pylori* on IL-8 production by AGS cells, AGS cells were incubated with *C. concisus* strains P2CDO4 and P3UCO1 at MOI 100 along with *H. pylori* strain 26695 at MOI 10 and 100 for 24 h, respectively. The AGS cell culture supernatants were then collected to measure the concentration of IL-8 using commercially available ELISA kits (Invitrogen, CA, USA) in triplicates, following the manufacturer’s instructions.

### Caspase 3/7 assay

Measurement of caspase 3/7 activity was used to assess apoptotic activity in AGS cells induced by *C. concisus*, as described previously (Lee et al., 2021). In brief, AGS cells were seeded on a black-walled 96-well plate with transparent bottoms at a concentration of 1 × 10^5^ cells/well in F-12K/FBS/Antibiotics medium. After 24 h incubation, the cell culture medium was replaced with F-12K/FBS medium. The AGS cells were then incubated with the six *C. concisus* strains described above at MOI 100 for 24 h, with untreated AGS cells serving as the negative control. *H. pylori* strain 26695 was included at MOI 10 and 100 as the bacterial control. To examine the combined effects of *C. concisus* and *H. pylori* on the apoptotic activity of AGS cells, AGS cells were incubated with *C. concisus* strains P2CDO4 and P3UCO1 at MOI 100 along with *H. pylori* strain 26695 at MOI 10 and 100 for 24 h, respectively. AGS cells were washed three times with Dulbecco’s phosphate-buffered saline (DPBS) before being stained with CellEvent Caspase-3/7 Green ReadyProbes reagent (Invitrogen), following manufacturer’s instructions. The fluorescence readings of caspase 3/7 activity were measured in triplicates and expressed in fold change relative to the untreated control.

### Examination of the effects of *C. concisus* on F-actin arrangement in AGS cells by fluorescence staining

AGS cells were seeded on coverslips in a 24-well plate at a concentration of 1 × 10^6^ cells/well in F-12K/FBS/Antibiotics medium. After 24 h incubation, the cell culture medium was replaced with F-12K/FBS medium. AGS cells were then incubated with *C. concisus* strains P2CDO4, P3UCO1, or *H. pylori* strain 26695 at MOI 100 for 24 h, with untreated AGS cells serving as the negative control. The AGS cells were fixed with 3.6% paraformaldehyde for 15 min, permeabilized with 0.1% triton for 10 min, and blocked with 1% bovine serum albumin (BSA) for 1 h. The filamentous actin (F-actin) and nuclei were then stained with Alexa Fluor 488 phalloidin (8878S, Cell Signaling Technology, MA, USA) and Hoechst 33342 (Invitrogen) respectively. The cells were mounted onto glass slides with 50% glycerol in water and examined using a fluorescent microscope (Olympus BX61; Olympus, Tokyo, Japan) with FITC (Excitation wavelength: 480 nm; Emission wavelength: 520 nm) and DAPI (Excitation wavelength: 365 nm; Emission wavelength: 430 nm) filters under the 100X objective. AGS cells without bacteria served as the negative control, and AGS cells incubated with *H. pylori* served as the positive control (Chang et al., 2016).

### Examination of the global gene responses induced by *C. concisus* in AGS cells by transcriptomic analysis

AGS cells were seeded in triplicates on 6-well cell culture plates at a concentration of 2 × 10^6^ cells/well in F-12K/FBS/Antibiotics medium. After 24 h incubation, the medium was replaced with F-12K/FBS medium. The AGS cells were then incubated with *C. concisus* strain P2CDO4, which was randomly selected.

Supernatants from AGS cells incubated with *C. concisus* strain P2CDO4 at MOI 50 for 4 h and without bacterial infection were collected for IL-8 measurement, as described above. The AGS cells were then washed three times with DPBS before being collected for RNA extraction. The total RNA of AGS cells was extracted using the ISOLATE II RNA Mini Kit (cat. no. BIO-52072; Bioline, NSW, Australia), following the manufacturer’s instructions. The purity and concentration of the extracted total RNA were measured using a NanoDrop spectrophotometer. The extracted total RNA was then submitted to the Ramaciotti Centre for Genomics, University of New South Wales, for RNA sequencing. The library preparation was conducted as previously described (Lee et al., 2023).

For RNA-seq data analysis, the raw RNA-seq reads were first checked for quality using FastQC (version 0.11.8). Adapters and low-quality reads were then trimmed using Trimmomatic (version 0.38) with the leading and trailing filters set to a minimum of Phred score 3 and a sliding window of 4:15, filtered reads with length less than 30 bp were also removed (Bolger et al., 2014). The trimmed reads were mapped against the human reference genome GRCh38.p14 using HISAT2 (version 2.1.0) under default settings (Kim et al., 2019). The mapped read counts SAM files generated from HISAT2 were then converted into BAM files using SAMtools (version 1.11) (Danecek et al., 2021). The mapped read counts were then quantified using featureCounts under the Subread package (version 2.0.1) for DEG analysis (Liao et al., 2014). Significantly differentially expressed genes between AGS cells with and without *C. concisus* infection were identified using the BioConductor package DESeq2 (version 1.36.0) in the R programming environment under default normalization methods, with adjusted *P* < 0.05 and log_2_ fold change < -1 and >1 being considered significant (Love et al., 2014).

### Gene ontology (GO) enrichment analysis

The list of differentially expressed genes (DEG) was uploaded to Metascape for GO enrichment analysis under default settings (Zhou et al., 2019). Enriched clusters of cellular biological processes were sorted according to *P*-value.

### Quantitative real-time PCR (qRT-pCR)

A literature search in the PubMed database was conducted to examine whether the upregulated genes in AGS cells, as revealed by transcriptomic analysis, were associated with the development of gastric cancer, using the gene name and gastric cancer as keywords. The upregulation of the gastric cancer-associated gene, *CYP1A1*, was further confirmed using qRT-PCR. For qRT-PCR, the total RNA (2 μg/sample) extracted from AGS cells with or without *C. concisus* infection was subjected to cDNA synthesis using the Tetro cDNA Synthesis kit (Bioline, NSW, Australia), following manufacturer’s instructions. SensiFAST SYBR No-ROX Mix (Bioline, NSW, Australia) was used for quantifying the synthesized cDNA in qRT-PCR in triplicates. The mRNA expression levels were normalized to the levels of the housekeeping gene glyceraldehyde 3-phosphate dehydrogenase (GAPDH) and expressed as fold changes relative to untreated cells, using the comparative threshold cycle CT (2^–ΔΔ*CT*^) method (Livak and Schmittgen, 2001). The sequences of PCR primers for quantification of CYP1A1 and qRT-PCR conditions are in Supplementary Table 2.

### Analysis of the role of CYP1A1 in gastric cancer patient survival

The gene encoding CYP1A1 was subjected to survival analysis in gastric cancer patients using the Kaplan–Meier Plotter website (Lánczky and Gyõrffy, 2021). The survival plot was generated using JetSet best probe set for the submitted genes, with a database of 631 gastric cancer patients which are classified as low or high expression cohorts. The overall survival differences between low and high expression cohorts were analyzed using the Kaplan-Meier method and log-rank test, *P* < 0.05 was considered as significant and other options remained default.

### Summary of experimental workflow

**FIGURE 1:**
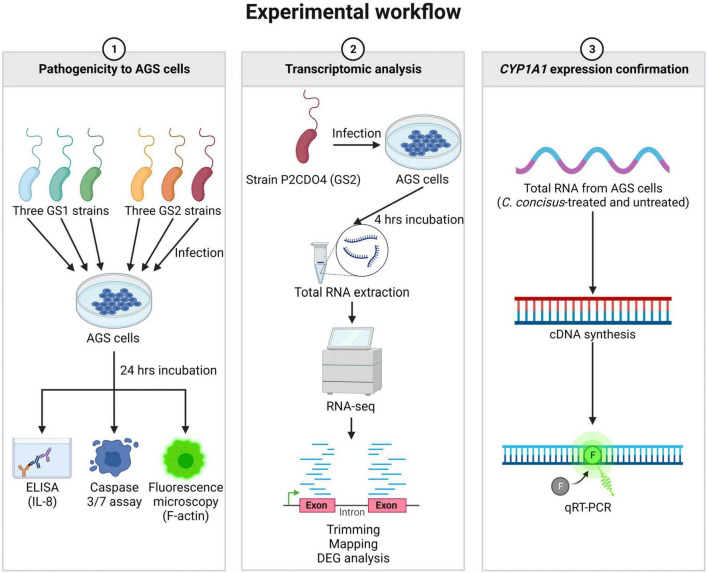
Summary of experimental workflow of this study. Experiments conducted in this study were classified into three categories. The first category involved examining the pathogenicity of *C. concisus* in AGS cells using ELISA, caspase 3/7 assay, and fluorescence microscopy. The second category focused on transcriptomic analysis of global gene response in AGS cells incubated with *C. concisus* through RNA-seq. The third category aimed to validate the gene expression level of *CYP1A1* using qRT-PCR. Figure created with BioRender.com.

The experimental workflow of this study is summarized in Figure 1, outlining the key experiments conducted. These include the examination of *C. concisus* pathogenicity to AGS cells, transcriptomic analysis of gene expression changes, and the subsequent validation of *CYP1A1* gene expression through qRT-PCR.

### Statistical analysis

*P*-values for different samples in ELISA and caspase 3/7 assay were calculated using one-way analysis of variance (ANOVA) with Dunnett’s test, while *P*-value for samples in qRT-PCR were calculated using two-tailed unpaired *t*-test. *P* < 0.05 was considered statistically significant. All statistical analyses were conducted using GraphPad Prism (version 9.5.1).

## Results

### *C. concisus* induced IL-8 production in AGS cells

**FIGURE 2:**
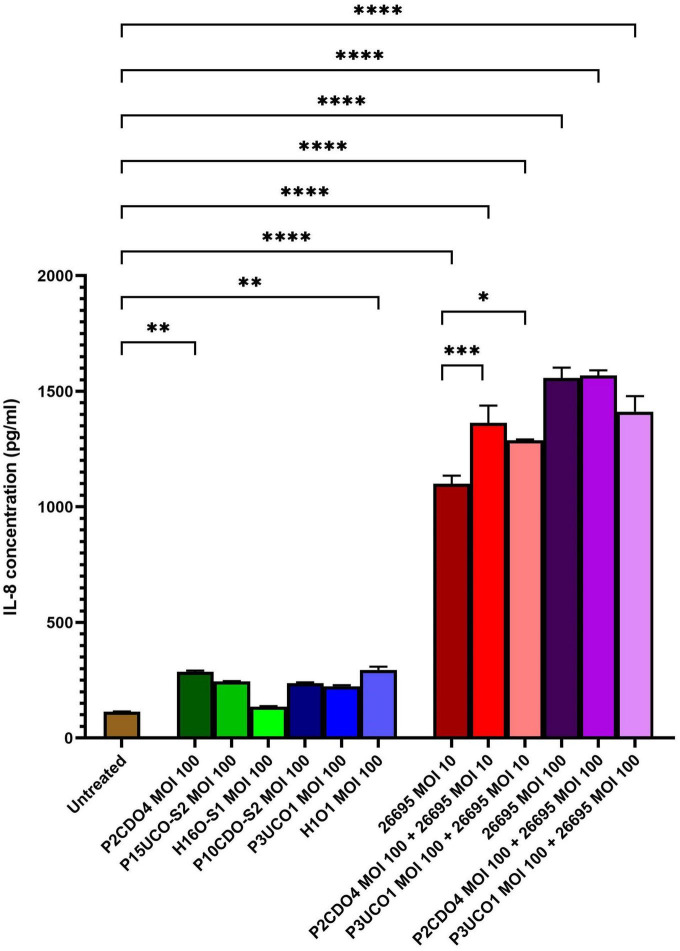
IL-8 production by AGS cells induced by *C. concisus* strains and *H. pylori* strain 26695 after 24 h incubation. IL-8 concentrations were measured by ELISA using supernatants of AGS cells after incubating with *C. concisus* strains and *H. pylori* strain 26695 for 24 h. *C. concisus* strains P2CDO4 and H1O1 at MOI 100 induced significantly higher IL-8 productions in AGS cells as compared to the untreated control (*P* < 0.01). *H. pylori* strain 26695 at both MOI 10 and 100 induced significantly higher IL-8 productions as compared to *C. concisus* at MOI 100. Co-incubation of *H. pylori* at MOI 10 with *C. concisus* strains (P2CDO4 and P3UCO1) induced significantly higher levels of IL-8 production as compared to that incubated by *H. pylori* alone (*P* < 0.001 and *P* < 0.05 respectively), however this increase was not observed when *H. pylori* was incubated at MOI 100. One-way analysis of variance (ANOVA) with Dunnett’s test was performed to test for statistical significance between untreated and infected AGS cells. Graph columns represent averages of triplicate experiments ± standard error (****P < 0.0001, ***P < 0.001, **P < 0.01, *P < 0.05; MOI, multiplicity of infection).

All six *C. concisus* strains examined at MOI 100 induced the production of IL-8 by AGS cells after a 24-h incubation period. Notably, the levels of IL-8 production in AGS cells incubated with *C. concisus* strains P2CDO4 and H1O1 were 286.64 ± 4.75 and 295.29 ± 11.5 pg/ml, respectively (*P* < 0.01). These values were statistically significant, indicating a higher induction of IL-8 production compared to AGS cells without bacterial infection (114.63 ± 0.68 pg/ml) (Figure 2). IL-8 production by AGS cells incubated with *C. concisus* strains P15UCO-S2, H16O-S1, P10CDO-S2, and P3UCO1 were 244.9 ± 1.22, 135.19 ± 2.41, 237.2 ± 3.12, and 224.54 ± 3.92 pg/ml, respectively. While these values were higher than the untreated sample, they were not considered statistically significant (*P* > 0.05) (Figure 2). Furthermore, *H. pylori* strain 26695 induced higher IL-8 production in AGS cells compared to *C. concisus* strains. The levels of IL-8 production by AGS cells incubated with *H. pylori* strain 26695 at MOI 10 and 100 were 1099.58 ± 29.18 and 1557.94 ± 36.05 pg/ml, respectively, which were significantly higher than the IL-8 production induced by the *C. concisus* strains (*P* < 0.0001) (Figure 2).

In AGS cells incubated with *H. pylori* strain 26695 at MOI 10, the presence of *C. concisus* increased the production of IL-8. Co-incubation of *H. pylori* strain 26695 with P2CDO4 and P3UCO1 resulted in significantly higher levels of IL-8 compared to AGS cell incubated with *H. pylori* alone, with concentrations of 1363.63 ± 60.97 (*P* < 0.001) and 1287.99 ± 3.47 pg/ml (*P* < 0.05), respectively (Figure 2). However, in AGS cells incubated with *H. pylori* strain 26695 at MOI 100, co-incubation with *C. concisus* strains P2CDO4 and P3UCO1 induced IL-8 production at 1568.5 ± 18.48 and 1412.19 ± 55 pg/ml, respectively, which was not significantly different from that induced by *H. pylori* alone (*P* > 0.05) (Figure 2).

### *C. concisus* induced apoptosis in AGS cells

**FIGURE 3:**
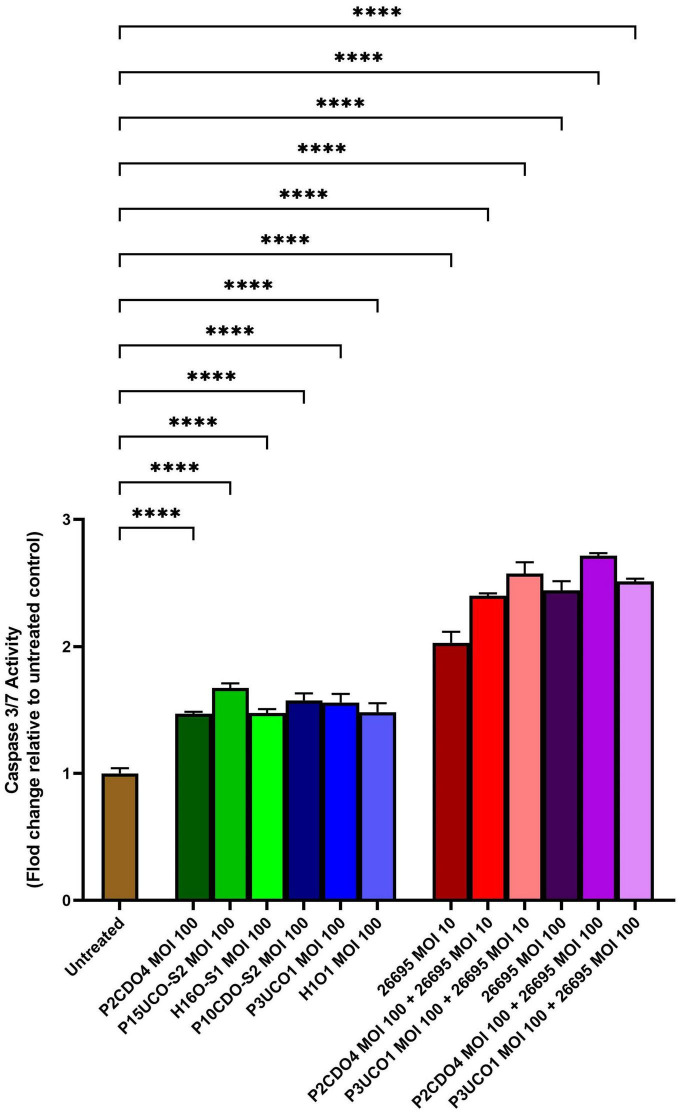
Caspase 3/7 activity of AGS cells infected with *C. concisus* strains and *H. pylori* strain 26695 after 24 h. Caspase 3/7 activity was measured using CellEvent caspase 3/7 green detection reagent. All six *C. concisus* strains at MOI 100, as well as *H. pylori* strain 26695 at both MOI 10 and 100, induced significantly higher levels of caspase 3/7 activity as compared to the untreated control (*P* < 0.0001). Co-incubation of *C. concisus* with *H. pylori* did not induce higher levels of caspase 3/7 activity as compared to that incubated with *H. pylori* alone. One-way analysis of variance (ANOVA) with Dunnett’s test was performed to test for statistical significance between untreated and infected AGS cells. Graph columns represent averages of triplicate experiments ± standard error (****P < 0.0001; MOI, multiplicity of infection).

*C. concisus* induced apoptosis in AGS cells when incubated with *C. concisus* strains at MOI 100 for 24 h, as indicated by increased caspase 3/7 activity. The levels of caspase 3/7 activity (expressed as fold change relative to the untreated AGS cells) in AGS cells incubated with *C. concisus* strains P2CDO4, P15UCO-S2, H16O-S1, P10CDO-S2, P3UCO1, and H1O1 at MOI 100 were 1.47 ± 0.01, 1.67 ± 0.03, 1.48 ± 0.02, 1.58 ± 0.04, 1.56 ± 0.06, and 1.48 ± 0.06 fold, respectively (*P* < 0.0001) (Figure 3). These values were all significantly higher than that of the untreated AGS cells. The caspase 3/7 activity in AGS cells incubated with *H. pylori* strain 26695 at MOI 10 and 100 for 24 h was 2.03 ± 0.07 and 2.44 ± 0.06 fold, respectively (*P* < 0.0001), indicating a higher apoptotic activity compared to that induced by *C. concisus* strains. Moreover, AGS cells incubated with *C. concisus* strain P2CDO4 at MOI 100, and P3UCO1 at MOI 100 along with *H. pylori* strain 26695 at MOI 10 and 100 respectively, also exhibited significantly higher apoptotic activity at 2.4 ± 0.02, 2.57 ± 0.07, 2.71 ± 0.02, and 2.51 ± 0.02 fold, respectively (*P* < 0.0001).

### *C. concisus* induced F-actin aggregation in AGS cells

**FIGURE 4:**
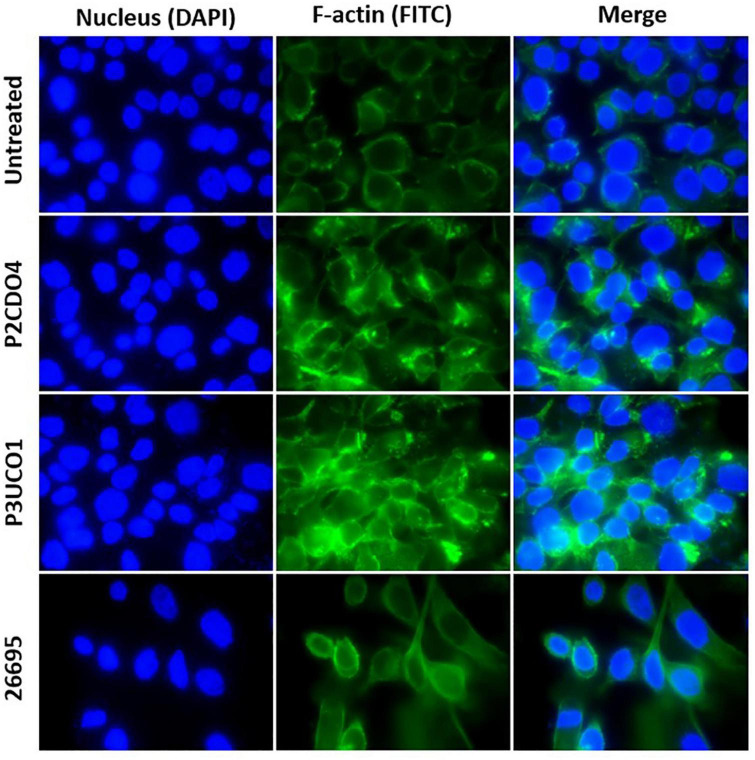
F-actin arrangement in AGS cells incubated with *C. concisus* strain P2CDO4, P3UCO1, and *H. pylori* strain 26695 for 24 h. Cell nucleus and F-actin were stained with Hoechst 33342 and Alexa Fluor 488 phalloidin and visualized using DAPI and FITC filters, respectively. Infection of AGS cells with the *C. concisus* strains P2CDO4 and P3UCO1 showed F-actin aggregation. *H. pylori* infection caused an elongated cell phenotype of AGS cells.

Both *C. concisus* strains examined caused F-actin aggregation in comparison to AGS cells without bacterial infection (Figure 4). AGS cells incubated with *H. pylori* strain 26695 showed sign of elongation, indicative of the hummingbird phenotype caused by the protein encoded by cytotoxin-associated gene A (CagA) in *H. pylori* (Moese et al., 2004; Chang et al., 2016).

### *C. concisus* upregulated the expression of gastric cancer associated CYP1A1 gene in AGS cells

**FIGURE 5:**
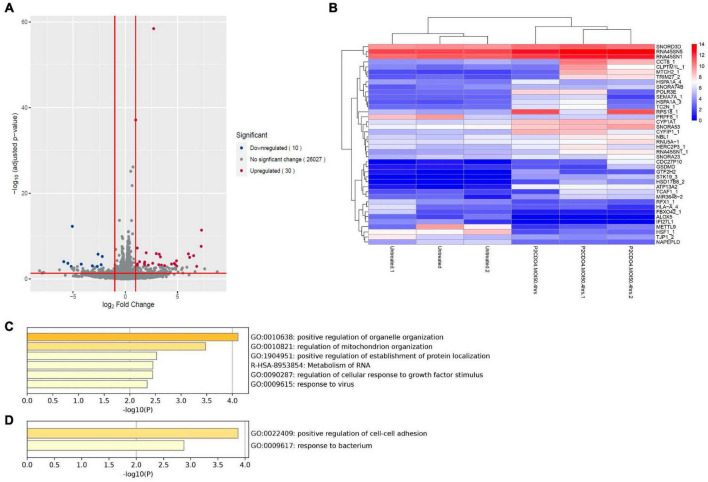
Transcriptomic analysis on global gene expression in AGS cells induced by *C. concisus* strain P2CDO4 after 4 h incubation. **(A)** The volcano plot illustrates a total of 26,067 transcripts from RNA-seq reads, with 30 transcripts significantly upregulated (red) and 10 transcripts significantly downregulated (blue). Genes with adjusted *P*-value < 0.05 and log_2_ fold change < -1 and >1 were considered statistically significant. Volcano plot generated using the EnhancedVolcano package on the R platform. **(B)** The heatmap shows the 40 differentially expressed genes based on log_2_ gene counts. Heatmap generated using the pheatmap package on the R platform. **(C)** The Gene ontology (GO) enrichment analysis revealed that upregulated genes mainly affect the regulation of organelle and mitochondria organization. **(D)** The GO enrichment analysis revealed that downregulated genes mainly affect the regulation of cell-cell adhesion.

Analysis of RNA-seq data revealed notable alterations in global gene response in AGS cells incubated with *C. concisus* strain P2CDO4 for 4 h, compared to untreated AGS cells (Supplementary Figure 1). From the global gene response, 40 genes were considered significantly altered in expression (adjusted *P*-value < 0.05, log_2_ fold change < -1 and >1), of which 30 genes were upregulated and 10 genes downregulated (Figures 5A, B and Supplementary Table 3). The upregulated genes are predicted to predominately affect organelle and mitochondrion organization, and the down regulated genes mainly affect the regulation of cell-cell adhesion (Figures 5C, D). Of the 30 upregulated genes by *C. concisus*, four genes were reported to be associated with gastric cancer including *HSD17B8_2*, *TRIM27_2*, *TC2N_1*, and *CYP1A1*. However, three of these genes had an underscore following their gene names, showing that they are on alternate reference locus (Bruford et al., 2020). We therefore decided to further characterize only *CYP1A1* gene using qRT-PCR.

**FIGURE 6:**
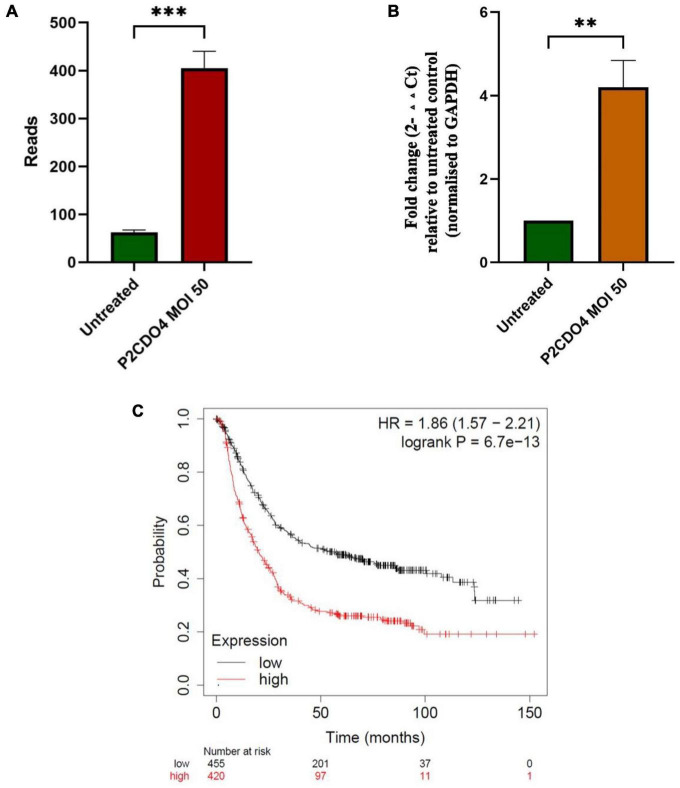
*C. concisus* strain P2CDO4 upregulated expression of gastric cancer associated *CYP1A1* gene in AGS cells. **(A)** The upregulation of the *CYP1A1* gene induced *by C. concisus* P2CDO4 was shown by gene reads from RNA-seq (***P < 0.001). **(B)** The upregulation of the *CYP1A1* gene was confirmed by qRT-PCR, with the experimental samples being AGS cells incubated with *C. concisus* strain P2CDO4 at MOI 50 for 4 h, and the untreated AGS cells as negative control. Fold change is calculated using the comparative threshold cycle CT (2^–ΔΔ*CT*^) method. Target gene fold change is relative to the untreated control, normalized to housekeeping gene GAPDH. Graph columns represent averages of triplicate experiments ± standard error (**P < 0.01; MOI, multiplicity of infection). **(C)** The survival plot shows that a higher level expression of CYP1A1 is associated with poor prognosis in gastric cancer patients, with a hazard ratio (HR) of 1.86 and median survival period of 20.4 months as opposed to 56.9 months in low expression cohort.

Transcriptomic analysis revealed that the expression of the upregulated gene *CYPIA1* was 6.57 fold in AGS cells incubated with *C. concisus* P2CDO4 at MOI 50 for 4 h, with a read count of 342.7 ± 35.38, compared to 62.3 ± 5.17 reads in untreated AGS cells (Figure 6A). This was confirmed using by qRT-PCR, which showed a 4.2 ± 0.64 fold increase relative to AGS cells without *C. concisus* infection (Figure 6B). According to the Kaplan-Meier Plotter survival analysis, a higher level expression of *CYP1A1* is associated with poor prognosis in gastric cancer patients, with a hazard ration of 1.86 and median survival period of 20.4 months as opposed to 56.9 months in low expression cohort (Figure 6C).

The IL-8 level was 199.96 ± 1.58 pg/ml in the supernatant of AGS cells incubated with *C. concisus* strain P2CDO4 for 4 h, and 179.83 ± 6.02 pg/ml in the supernatant of untreated AGS cells (*P* < 0.005).

The RNA-seq data have been deposited in NCBI’s Gene Expression Omnibus and are accessible through GEO Series accession number GSE242316.^1^

## Discussion

This study aimed to investigate the pathogenic effects of *C. concisus* strains on human gastric epithelial cells using AGS cells as a model for gastric epithelium.

*C. concisus* strains were found to induce the production of proinflammatory cytokine IL-8 in AGS cells following 24 h of incubation. IL-8, a chemokine known for inducing chemotaxis of neutrophils and other immune cells (Harada et al., 1994). In our study, all *C. concisus* strains induced the production of IL-8 in AGS cells, suggesting that *C. concisus* has the potential to induce gastric inflammation. The significant genomic diversity observed among *C. concisus* strains aligns with their varying capacities to induce IL-8 (Figure 2; Gemmell et al., 2018; Aagaard et al., 2021). *C. concisus* strains induced much less gastric epithelial production of IL-8, as compared to *H. pylori* strain 26695 (Figure 2). This finding suggests that *C. concisus* bacteria are more inclined to cause mild gastric inflammation in comparison to the more robust inflammatory response triggered by *H. pylori*.

In a study by Ma et al. (2015) it was observed that 4 out of 20 *C. concisus* strains were able to survive a 30-min exposure to low pH in a test tube. This suggests that certain *C. concisus* bacteria may endure the gastric environment for a limited period, potentially allowing them to relocate to a safer zone (Ma et al., 2015). In the human stomach, the mucus layer proximal to the epithelial cells maintains a higher pH. Given the motility and spiral to curved shape of *C. concisus* bacteria, it is conceivable that they could swim toward a more secure region closer to the gastric epithelium, similar to *H. pylori*. This ability to survive in the gastric environment aligns with findings by Ferreira et al. (2022) who cultured *C. concisus* from gastric biopsies, providing further evidence for the potential colonization of the human stomach by *C. concisus* bacteria. However, as *C. concisus* does not possess urease, their ability in resisting gastric acid would be lower than *H. pylori*.

Interestingly, we found that co-infection of *C. concisus* and *H. pylori* increased the production of IL-8 in AGS cells, suggesting a potential synergistic effect between these bacteria in inducing inflammation. This could have implications for individuals with gastric *H. pylori* colonization, where the presence of *C. concisus* might enhance the inflammatory response in gastric epithelial cells. On the other hand, *C. concisus* did not increase IL-8 production in AGS cells incubated with *H. pylori* at MOI 100, most likely due to that the AGS cells have reached its maximum capability of producing IL-8 under the stimulation of higher numbers of *H. pylori* bacteria (Figure 2).

The increase in caspase 3/7 activities in AGS cells following 24 h incubation with *C. concisus* strains were significant, although *H. pylori* strain 26695 induced a significantly higher level of caspase 3/7 activity than *C. concisus* (Figure 3). This suggests that *H. pylori* has caused more damage to gastric epithelial cells than *C. concisus* under the same MOI, which apoptotic activity in gastric epithelial cells was known to increase in *H. pylori*-induced gastritis (Dang et al., 2020). The co-infection of *C. concisus* and *H. pylori* further increased the level of caspase 3/7 activity in AGS cells, suggesting a potential synergistic effect on apoptosis (Figure 3). Our findings suggest that induction of apoptosis is a potential mechanism by which *C. concisus* may contribute to gastric diseases, especially when coexisting with *H. pylori*.

*C. concisus* induced F-actin aggregation in AGS cells. The actin cytoskeleton regulates various cellular processes in eukaryotic cells, providing structural integrity at cell-cell junctions and promote membrane extensions (Hartsock and Nelson, 2008; Svitkina, 2018). Actin filaments are frequently targeted by bacterial pathogens, including *H. pylori*. *H. pylori* adheres to gastric epithelial cells via adhesins BabA and SabA and delivers CagA and other bacterial factors into the host epithelial cytosol. This process results in the phosphorylation and dephosphorylation of proteins in different pathways, rearrangement of actin and ZO-1, and disruption of the gastric epithelial barrier. Previous studies have reported that *H. pylori* CagA can induce a “hummingbird” phenotype change in gastric epithelial cells, which was also observed in our experiment (Figure 4). Interestingly, we found that *C. concisus* caused a different type of actin rearrangement, specifically actin aggregations (Figure 4). The virulence factors of *C. concisus* that affect the epithelial actin arrangement and their mechanisms are not clear, warranting further investigation in future studies.

*C. concisus* bacteria transported from the oral cavity to the stomach through swallowed saliva may not establish long-term gastric colonization. To investigate potential changes in gene expression after short-term contact with gastric epithelial cells, we investigated the global gastric epithelial gene responses to *C. concisus* using transcriptomic analysis following a 4-h incubation at a lower dose (MOI 50). Global gene response in AGS cells incubated with *C. concisus* strain P2CDO4 for 4 h was altered when compared to the untreated AGS cells (Supplementary Figure 1), of which a total of 30 genes and 10 genes were significantly upregulated and downregulated, respectively, which were predicted to affect several cellular functional pathways (Figure 5 and Supplementary Table 3). These findings suggests that *C. concisus* bacteria passing through the stomach from the oral cavity may exert regulatory effects on gastric epithelial cell gene expression. We further confirmed the upregulation of expression of the gene coding CYP1A1 through qRT-PCR. CYP1A1, a cytochrome P450 enzyme involved in metabolism of xenobiotics (Androutsopoulos et al., 2009), has been associated with gastric cancer and precancerous gastric cancer as well as lung cancer (Kouri et al., 1982; Zhang et al., 2004; Hidaka et al., 2016; Huang et al., 2021; Sadeghi-Amiri et al., 2021). Previous research by Cui et al. (2019) linked the abundance of *C. concisus* in tongue coating microbiome to gastric precancerous cascade, which aligns with our finding of *C. concisus* upregulating CYP1A1 expression. This provides additional evidence that oral *C. concisus* may play a role in facilitating gastric cancer development or progression. This is further supported by the survival analysis of CYP1A1 in gastric cancer patients (Figure 6).

In summary, our study reveals that incubation of *C. concisus* with human gastric epithelial cells (AGS cells) induces the production of IL-8, apoptosis, and upregulation of CYP1A1 gene expression in gastric epithelial cells. These findings suggest that *C. concisus* may play a role in gastric inflammation and the progression of gastric cancer. Further investigation in clinical studies is warranted.

## Data availability statement

The datasets presented in this study can be found in online repositories. The names of the repository/repositories and accession number(s) can be found in this article/Supplementary material.

## Ethics statement

Ethical approval was not required for the studies on humans in accordance with the local legislation and institutional requirements because only commercially available established cell lines were used.

## Author contributions

CL: Formal analysis, Writing – original draft, Investigation. SL: Methodology, Writing – review and editing. NN: Writing – review and editing, Investigation. FL: Methodology, Writing – review and editing. AT: Funding acquisition, Writing – review and editing. LW: Writing – review and editing, Conceptualization, Funding acquisition. SR: Writing – review and editing, Conceptualization. LZ: Writing – review and editing, Conceptualization, Funding acquisition, Supervision.

## Footnotes

1
https://www.ncbi.nlm.nih.gov/geo/query/acc.cgi?acc=GSE242316

## Supplementary material

Supplementary Figure 1 Principal component analysis (PCA) plot of RNA-seq data. A total of 6 samples were included in the analysis, red dots for treatment with *C. concisus* strain P2CDO4 and blue dots for untreated control. The plot indicates separation between control and treatment groups, suggesting significant changes in overall gene response in AGS cells upon *C. concisus* infection. Gene counts were normalized using log2 transformation. PCA plot generated using the ggplot2 package on the R platform.
