# Supplementary figures and images for: Investigation of Campylobacter concisus gastric epithelial pathogenicity using AGS cells
Source: Front Microbiol. 2024 Jan 11;14:1289549. doi: 10.3389/fmicb.2023.1289549 (PMC10808343; doi:10.3389/fmicb.2023.1289549)

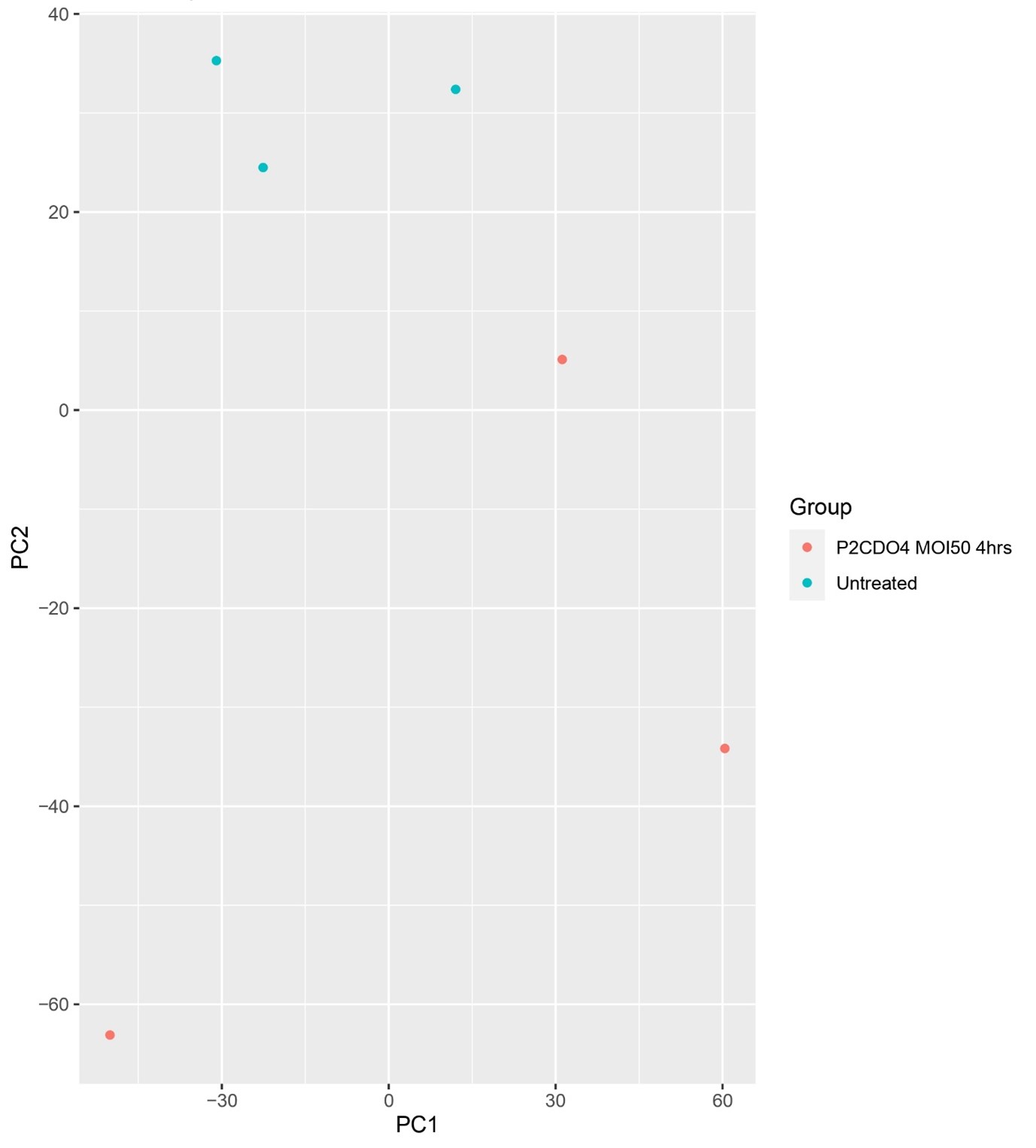

Supplement: Supplementary Figure 1 — Principal component analysis (PCA) plot of RNA-seq data. A total of 6 samples were included in the analysis, red dots for treatment with C. concisus strain P2CDO4 and blue dots for untreated control. The plot indicates separation between control and treatment groups, suggesting significant changes in overall gene response in AGS cells upon C. concisus infection. Gene counts were normalized using log2 transformation. PCA plot generated using the ggplot2 package on the R platform. [file Image_1.JPEG]
