# Supplementary material for: Investigation of Campylobacter concisus gastric epithelial pathogenicity using AGS cells
Source: Front Microbiol. 2024 Jan 11;14:1289549. doi: 10.3389/fmicb.2023.1289549 (PMC10808343; doi:10.3389/fmicb.2023.1289549)
Supplement: Supplementary file 2 [file Table_1.DOCX]

**Supplementary Table 1 Campylobacter concisus strains used in this study**

| Strain | GS | Isolation source | Health status | Assembly accession no. | Reference |
| --- | --- | --- | --- | --- | --- |
| P10CDO-S2 | 1 | Oral cavity | CD | CP049274 | [1] |
| P3UCO1 | 1 | Oral cavity | UC | CP049239 | [1] |
| H1O1 | 1 | Oral cavity | Healthy | CP049237 | [1] |
| P2CDO4 | 2 | Oral cavity | CD | CP021642-CP021643 | [1] |
| P15UCO-S2 | 2 | Oral cavity | UC | CP049234-CP049236 | [1] |
| H16O-S1 | 2 | Oral cavity | Healthy | CP049263 | [1] |

GS: Genomospecies; CD: Crohn’s disease; UC: Ulcerative colitis

**Reference**

[1] Liu F, Ma R, Tay CYA, et al. Genomic analysis of oral Campylobacter concisus strains identified a potential bacterial molecular marker associated with active Crohn’s disease. Emerging microbes & infections. 2018;7:1-14.
