# Supplementary material for: Investigation of Campylobacter concisus gastric epithelial pathogenicity using AGS cells
Source: Front Microbiol. 2024 Jan 11;14:1289549. doi: 10.3389/fmicb.2023.1289549 (PMC10808343; doi:10.3389/fmicb.2023.1289549)
Supplement: Supplementary file 3 [file Table_2.DOCX]

Supplementary Table 2 Primers used for qRT-PCR

| Target gene | Forward sequence  (5’ 🡪 3’) | Reverse sequence  (5’ 🡪 3’) | Product size (bp) | Reference |
| --- | --- | --- | --- | --- |
| CYP1A1 | GATTGAGCACTGTC  AGGAGAAGC | ATGAGGCTCCAGGAG  ATAGCAG | 138 | [1] |
| GAPDH | GAGTCAACGGATTT  GGTCGT | TTGATTTTGGAGGGA  TCTCG | 238 | [1] |

qRT-PCR conditions: holding temperature of 95^o^C for 2 min, cycling condition of 95^o^C for 10 seconds, 60^o^C for 15 seconds, 72^o^C for 20 seconds for 40 cycles. Melting temperature of 35^o^C - 95^o^C.

**Reference:**

[1] Ye J, Coulouris G, Zaretskaya I, et al. Primer-BLAST: a tool to design target-specific primers for polymerase chain reaction. BMC bioinformatics. 2012;13:1-11.
